# Peer-assisted learning: time for nomenclature clarification

**DOI:** 10.3402/meo.v21.30974

**Published:** 2016-07-12

**Authors:** Alexander Olaussen, Priya Reddy, Susan Irvine, Brett Williams

**Affiliations:** 1 Department of Community Emergency Health and Paramedic Practice, Monash University, Melbourne, Australia; 2 Emergency & Trauma Centre, The Alfred Hospital, Melbourne, Australia; 3 National Trauma Research Institute, The Alfred Hospital, Melbourne, Australia

**Keywords:** PAL, peer-assisted learning, near-peer, mentor, tutoring, didactic, near-peer teacher

## Abstract

**Background:**

Peer-assisted learning (PAL) is used throughout all levels of healthcare education. Lack of formalised agreement on different PAL programmes may confuse the literature. Given the increasing interest in PAL as an education philosophy, the terms need clarification. The aim of this review is to 1) describe different PAL programmes, 2) clarify the terminology surrounding PAL, and 3) propose a simple pragmatic way of defining PAL programmes based on their design.

**Methods:**

A review of current PAL programmes within the healthcare setting was conducted. Each programme was scrutinised based on two aspects: the *relationship* between student and teacher, and the student to teacher *ratio*. The studies were then shown to fit exclusively into the novel proposed classification.

**Results:**

The 34 programmes found, demonstrate a wide variety in terms used. We established six terms, which exclusively applied to the programmes. The relationship between student and teacher was categorised as peer-to-peer or near-peer. The student to teacher ratio suited three groupings, named intuitively ‘Mentoring’ (1:1 or 1:2), ‘Tutoring’ (1:3–10), and ‘Didactic’ (1:>10). From this, six novel terms – all under the heading of PAL – are suggested: ‘Peer Mentoring’, ‘Peer Tutoring’, ‘Peer Didactic’, ‘Near-Peer Mentoring’, ‘Near-Peer Tutoring’, and ‘Near-Peer Didactic’.

**Conclusions:**

We suggest herein a simple pragmatic terminology to overcome ambiguous terminology. Academically, clear terms will allow effective and efficient research, ensuring furthering of the educational philosophy.

Peer-assisted learning (PAL) as an educational method has been around since Socrates and Plato began questioning one another's ideas in small groups (1). In recent times, PAL has gained increasing attention across many different healthcare disciplines and educational sectors (1). Naturally following such is a growing body of evidence to determine its usefulness.

The benefits of PAL has been well-described by Topping et al. (1) and clearly pertain to all stakeholders (i.e., the universities, the peer-teacher, and the peer-learner) (1–3). There appears to be a climate of readiness to formally incorporate PAL into different areas of healthcare studies.

PAL has one philosophy: students learning from students (4). Two different relationships between the students and some variations in the arrangement of PAL programmes have carved out the different methods described to date. Given the simple and common root that all PAL programmes stem from, the extensively varying terminologies used is peculiar. Several mismatched terms exist throughout the literature. Examples of these include, but are not limited to: *peer-led teaching, peer-led training, peer-tutoring, peer-teaching, collaborative learning, collaborative tutoring, cooperative learning, supplementary instruction, tutor-less group, peer supported learning, shared learning, co-teaching, co-tutoring, student partnership, facilitated peer mentoring*, and similar variations of *near-peer* or *cross year*. The most commonly used term – peer-assisted learning – is arguably just an umbrella term encompassing all PAL programmes, and as such this term is non-descriptive (1).

PAL has been extensively researched in the pedagogy (5) and seems to carry less confusion about the terminology than in andragogy. This may be because adult learners are more heterogeneous than the young, as well as the environment in which they learn differs. The variance also appears in the preferred learning methods and the personal motivation (6). Adult learners’ ‘*richest resources for learning reside in the adult learners themselves*’ (6) (p. 45). Focus on experience-based techniques, including PAL, is therefore beneficial.

Terms need to be consistent for a number of reasons. Firstly, programme implementation is facilitated chiefly by clear terminology, communication, and intent. Secondly, for research purposes building an evidence-based foundation is more achievable. The uncertainty and incongruence around the terms weakens and confuses the research starting point. Thirdly, communication across institutions and disciplines is eased through accurate and consistent terms.

Attempts to clarify the terminology exist. Ladyshewsky (7) outlined different PAL methods and suggested groupings based on common ‘indices’. Ten Cate and Durning (8) designed a framework distinguishing between three elements.

Ladyshewsky (7) argues there are two common indices that can describe all methods of implementing PAL – namely, 1) equality (e.g., to which extent learners take direction from each other) and 2) mutuality (e.g., in relation to the learners’ discourse). Although this may be theoretically sound, its applicability is limited by the non-practical definitions. Moreover, a single PAL programme may be difficult to define within the suggested category, as the indices are not quantifiable as well as overlapping.

Ten Cate and Durning (8) on the other hand distinguish PAL programmes based on three believed core components: 1) education distance, 2) group size, and 3) formality. The distance is undoubtedly a key factor to consider and should differentiate between peers and near-peers. Further, the size of the group is also important as it has practical implications for the educational providers and correlates with students’ preferences and learning (9). There is limited evidence around the impact formality has on the PAL outcomes. Furthermore, this is difficult to include in nomenclature given the spectrum formal involvement lies on and its vast variation amongst different education institutions.

Despite these clarifying attempts, inconsistencies continue to exist throughout the literature. This may be because the suggested components are difficult to define. We therefore aim to 1) describe the different methods in which PAL programmes have been incorporated to date, 2) clarify the terminology surrounding PAL, and 3) propose a simple pragmatic way of defining PAL programmes.

## Methods

We searched five databases (PubMed, Cinahl, Medline, Proquest, and Embase) and two grey literature websites (www.greylit.org and www.tripdatabase.com), in a scoping review manner for articles of relevance (10). The articles were narrowed down based on the key concepts of describing the implementation of a PAL programme and pertaining to the healthcare education. We included studies describing different forms of PAL in order to i) show the wide and varied approach PAL can take, and ii) to ensure that our suggested novel terms would be applicable to all methods of PAL practice.

We derived the new terms from a consensus process stemming from the different PAL methodologies within the literature. In accordance with previous nomenclature clarification attempts within other fields, we desired to keep well-established acronyms where possible, whilst also clarifying any confusion through making the novel terms more accurate in their description (11).

## Results

We describe 34 different approaches to PAL. From the findings, a clear disparity in nomenclature was determined, further highlighting the importance of formalising the terminology around PAL. The 34 programmes reviewed are listed in Table 1. Their methods and used terminology are tabulated.

**Table 1:** An overview of different PAL programs, their method and used terminology, presented sequentially based on the novel terminology

Proposed terminology and the corresponding teacher-to-student ratio	Study title (reference)	Method	Suggested name by the study
**Peer-to-peer**			
Peer Mentoring (1 to 1–2)	Relationship between retention and peer tutoring for at-risk students (12)	26 ‘at-risk’ nursing students were randomised to a PAL or control group. 20 were given peer tutors (who had a higher academic score than the learner) in a one to one fashion.	‘Peer tutoring’
	Clay modelling for pelvic anatomy review for third-year medical and physician assistant students (13)	23 third year medical students taught one another female anatomy after listening to a lecture and seeing a demonstration.	‘Peer learning (peer learner and peer teachers)’
	Learning in the simulated setting: a comparison of expert-, peer-, and computer-assisted learning (14)	60 medical students were randomised to three groups. All were given a brief lecture. The peer group was split into groups of two where they taught each other while the other group consisted of computer-assisted learning.	‘PAL’
	Peer assisted learning in surgical skills laboratory training: a pilot study (15)	Residents taught each other with and without guidelines then provided feedback to each other on the skills practiced.	‘Peer feedback’ and ‘peer teaching’ was referred to as PAL
Peer Tutoring (1 to 3–10)	A controlled trial of peer-teaching in practical gross anatomy (16)	160 second year medical students, 80 of which were controls. Half the group would dissect then they would teach the next group then retire to study while the second group dissected. The second group then showed the first group.	‘Peer teaching’
	A comparison of learning outcomes and attitudes in student- versus faculty-led problem-based learning: an experimental study (17)	Second year medical students were assigned a peer within groups of 10 to facilitate tutorials.	‘Peer facilitator’
	Student-led tutorials in problem-based learning: educational outcomes and students’ perceptions (18)	Third year medical students taught each other in groups of 8–10.	‘Student led tutorials’
	Involvement in teaching improves learning in medical students: a randomized cross-over study (19)	135 first year medical students rotated the role of tutor and tutee in small groups with two tutors per group.	‘Peer educators’
	Knowledge transfer of spinal manipulation skills by student-teachers: a randomised controlled trial (20)	292 third and fourth year medical students were taught in groups of 6–12 by fellow peers (who received brief teaching course).	‘Student teachers’
	Peer teaching: a randomised controlled trial using student-teachers to teach musculoskeletal ultrasound (21)	151 students, 75 of which were taught by nine student teachers of the same year.	‘Student teachers’
Peer Didactic (1 to > 10)	Peer assisted versus expert assisted learning: a comparison of effectiveness in terms of academic scores (22)	70 fourth year medical students where one group (35 students) was given a lecture by a peer who had the highest academic score.	‘Reciprocal peer teaching’
**Near-Peer**			
Near-Peer Mentoring (1 to 1–2)	Reducing student anxiety by using clinical peer mentoring with beginning nursing students (23)	30 ‘freshmen’ nursing students were paired with individual ‘sophomore’-level medical-surgical peer mentors.	‘Peer mentoring’
Near-Peer Tutoring (1 to 3–10)	Can near-peer medical students effectively teach a new curriculum in physical examination? (24)	83 third year medical students were taught in groups by nine 4th/5th years.	‘Near peer teaching’
	Peer assisted learning in patient-centred interviewing: the impact on student tutors (25)	Two third year medical students taught groups of six first year medical students.	‘Student tutors’
	Student teachers can be as good as associate professors in teaching clinical skills (26)	Medical students in year two and above taught first year medical students in groups of 5–6.	‘Student teachers’
	Formal peer-teaching in medical school improves academic performance: the MUSC supplemental instructor program (27)	Medical students from upper levels taught junior students in groups of 4–6.	‘Supplemental instructors’
	Peer tutoring and student outcomes in a problem-based course (28)	Medical students who had completed a particular course two semesters prior taught current students in groups of 4–8.	‘Peer tutors’
	Advanced Cardiac Resuscitation Evaluation (ACRE): a randomised single-blind controlled trial of peer-led vs. expert-led advanced resuscitation training (29)	One sixth year medical student taught cardiac resuscitation to nine fifth year medical student.	‘Peer instructors’ & ‘Peer led training’
	Are fourth-year medical students effective teachers of the physical examination to first-year medical students? (30)	Nine fourth year medical students taught first year medical students in groups of four.	‘Student preceptor’
	Peer-assisted learning from three perspectives: student, tutor and co-ordinator (2)	Small group sessions with 12 students per two peer tutors. Peer tutors were generally one year senior. Consultants reviewed the teaching and learning material. Peer tutors received training in the relevant skills.	‘Peer tutors’ within a PAL framework
	Impact of peer teaching on nursing students: perceptions of learning environment, self-efficacy and knowledge (31)	179 first year nursing students were taught by 51 third year students.	‘Peer teaching’
	Peer-assisted learning in the acquisition of clinical skills: a supplementary approach to musculoskeletal system training (32)	Four fourth year medical students trained 28 second year students with 218 control students.	They called it ‘PAL’ but referred to the near peers as ‘student trainers’
	Undergraduate rheumatology: can peer-assisted learning by medical students deliver equivalent training to that provided by specialist staff? (33)	12 senior medical students trained 45 second	‘Student trainers for PAL’ year students.
	Randomized surgical training for medical students: resident versus peer-led teaching (34)	60 third year medical students taught by fourth years in groups of 4–5.	‘PAL’
	Peer-led resuscitation training for healthcare students: a randomised controlled study (35)	122 first year medical, dental, nursing and physiotherapy students taught by second years in groups of 10–12 with two peers (of 1 year higher) per group.	‘Student instructors’ in ‘peer led’ training
	Near-peer teaching in anatomy: an approach for deeper learning (36)	12 fourth year medical students ran dissection classes for first and second year students (no specific number was stated but it is noted that the entire first and	‘Near peer teachers’ abbreviation used ‘NP’ Students were called ‘tutees’
		second year class was involved in this programme). 2–3 ‘near-peer teachers’ were assigned to each small group.	
	Peer-assisted versus faculty staff-led skills laboratory training: a randomised controlled trial (37)	89 third year medical students divided into three groups of controls (28), PAL (run by fourth and fifth years) (29), and staff taught (26).	‘Cross year’ PAL
Near-Peer Didactic (1 to >10)	A vertical study programme for medical students: peer-assisted learning in practice (38)	Fifth year medical students provided five sets of 2 h case based lectures in groups of 10–15 medical students from years one to four.	‘PAL facilitators’
	Effects of peer-assisted training during the neurology clerkship: a randomized controlled study (39)	Six medical students who had completed a neurological clerkship the semester prior taught 66 medical students currently undertaking their clerkship.	‘Peer tutoring’
	A multi-level assessment of a program to teach medical students to teach (40)	28 fourth year medical students taught 117 second year medical students.	‘Student teachers’
	The role of students as teachers: four years’ experience of a large-scale, peer-led programme (41)	Eight medical students within their clinical phase taught 358 junior medical students in their pre-clinical phase.	‘Peer led teaching’ and the learners were referred to as ‘tutees’
	Peer-assisted learning: a novel approach to clinical skills learning for medical students (42)	Three year 4–5 students taught 86 year 1–2 students: one near-peer to 23 students, 1 to 29 students, and 1 to 34 students.	‘Trainer and Trainee’
	Clinical skills education: outcomes of relationships between junior medical students, senior peers and simulated patients (43)	125 second year medical students were trained by 11 sixth year students.	‘Cross year PAL’
	A three-day anatomy revision course taught by senior peers effectively prepares junior students for their national anatomy exam (44)	105 second year medical students taught by four fourth year students in a lecture setting.	‘Course tutors’
	Peer-assisted teaching: an interventional study (45)	One third year paramedic student taught 12 first year paramedic students with the presence of a paid sessional staff member.	‘Peer teaching’

**Fig. 1:**
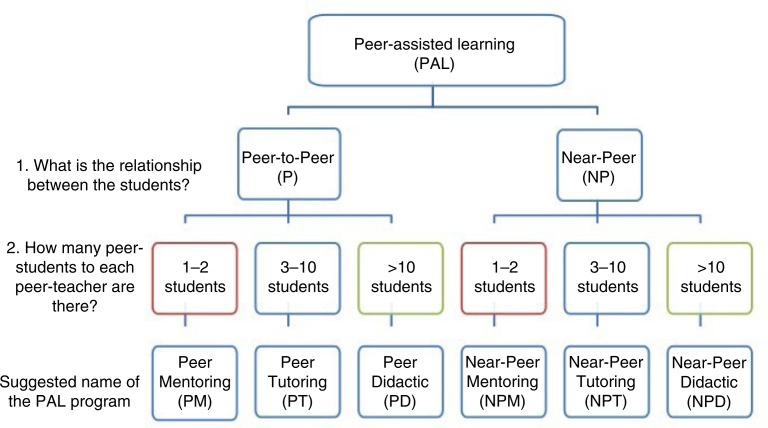
Categories of PAL and the novel terms.

Given the wide variety, we propose a new pragmatic indexing approach, which is based on unambiguous components. Based on components commonly used to describe the programmes, we propose the new classification relates to the *relationship* between the students and the *ratio* of students to student-facilitators (Fig. 1).

## Discussion

### The umbrella term: PAL

PAL is the umbrella term and encompasses all programmes in which students learn from students, and does not specify any more than that. There seems to be confusion in the literature between PAL as an umbrella term and peer-to-peer learning. Peer-to-peer is the appropriate name when the students are peers; as opposed to near-peers. Considering that both students (i.e., the teacher and the one being taught) are learning and benefitting (3, 4) may alleviate the confusion. Thus, the term peer-learner should not solely describe the student – which it often does – but rather describe both the teacher and student. We do not wish to alter this terminology because the acronym PAL is widely known and utilised.

#### Relationship between students: peer or near-peer

We consider peer and near-peer the first key separation because cognitive congruence is a vital component of learning (8).

Our proposed classification therefore immediately begs the question – what is a peer? Is a peer merely someone at the same academic year level, or is it more appropriate to distinguish based on ability? Whilst disagreement on this question flourishes in the literature, Ladyshewsky (7) and King (46) concludes that without pairing students’ status and ability, the programme becomes simply tutoring, not peer-tutoring. The role of the faculty will be in facilitating and monitoring the relationship between their students.

The definition of a near-peer is generally clearer, and consists of two participants that are at least one academic year apart. However, exceptions exist. For instance, when PAL is used within interdisciplinary programmes, (47) students may have different abilities although being at the same academic year level. We suggest that cases of interdisciplinary PAL programmes should be referred to as near-peers when they are at the same acedemic year level.

### Ratio of students: mentoring, tutoring, or didactic

In concordance with Ten Cate and Durning (8), we also consider the number of students in the group crucial. This correlates with students’ preferences (9), thereby affecting the likelihood of engagement and implicitly learning. Ten Cate and Durning (8) split the size of the PAL group into only two groups (i.e., 1 to <3 students, and 1 to ≥3 students). Given the varying dynamic of different group size, this distinction may be too blunt. We therefore propose a three-way split which is more consistent with traditional academic structuring, namely mentoring, tutoring, and didactic.

We define a programme as a mentor programme if the teacher to student's ratio is 1:1 or 1:2 (i.e., a microenvironment). Mentoring involves positive role modelling and reinforcement, supplemented by counselling, often used for disadvantaged groups (3). PAL by mentoring is beneficial in that it provides a more intimate setting where students are more inclined to ask questions and express uncertainties. Furthermore, the likelihood of student involvement in the process and direct monitoring of student progress by the teacher can be easily facilitated. The obvious drawback of PAL mentoring lies in resource demand. Finding compatible pairs is a challenge for the institution.

We define a tutorial as a setting where there is one teacher to between 3 and 10 students. Tutoring is often highly focused on curricula content and is characterised by the assignment of specific roles (i.e., tutor and tutee), most often with clear guidance around the structure (3). The benefits of peer-tutoring are i) less resource demanding, ii) increased possibility for the university to follow up their peer-teachers, and iii) raised and diversified collaboration given the larger group and the inherent broader range of views and perceptions. However, this also leads to the possible drawback that quiet students may remain quiet and unnoticed, thereby limiting the utility of such a PAL programme for those students.

We define a programme as didactic if the teacher to student's ratio is in excess of 1:10. Among the vast array of learning methods and styles, although less common in PAL, is a class delivered lecture format. This one directional method is beneficial in that it uses minimal resources and teaches the peer-teacher to both prepare and present in front of a large group. However, limited possibilities for feedback, participation, and student interaction are considerable drawbacks to this method.

Based on the above-described components, every PAL programme will fall, mutually exclusively, under any of six categories. Figure 1 outlines these categories and illustrate their corresponding suggested names.

## Conclusion

We have herein tabulated the main variations in PAL programmes and proposed a novel nomenclature classification. We are not classifying previous authors and their terminology as wrong, nor do we wish to correct them. We merely encourage future research in this field to be more consistent with its terminology. This will better enable the formal integration of PAL into educational programmes. To overcome the shortcomings of previous attempts at clarifying the terminology, we have proposed a clear, intuitive, and unambiguous nomenclature in which programmes mutually exclusively belong to just one term.

To broaden the platform of research around PAL and to allow easy integration across institutions, consistent terms and definitions are necessary. We urge consistent use of the PAL terms based on the suggested groupings offered in this paper. Expansion of the MeSH (Medical Subject Headings) terms is necessary. It may be anticipated that new terminology introduction may be inconvenient at first, and it is unlikely that a consensus will be reached quickly; however, we believe the long-term benefits uniform terminology has on research and education outweigh this hindrance.
